# Geographical origin of *Plasmodium vivax* in the Hainan Island, China: insights from mitochondrial genome

**DOI:** 10.1186/s12936-023-04520-7

**Published:** 2023-03-08

**Authors:** Yuchun Li, Xiaomin Huang, Ling Qing, Wen Zeng, Xiangjie Zeng, Feng Meng, GuangZe Wang, Yan Chen

**Affiliations:** Hainan Provincial Centre for Disease Control and Prevention, Haikou, 570203 China

**Keywords:** *Plasmodium vivax*, Mitochondrial genome, Hainan

## Abstract

**Background:**

Hainan Province, China, has been an endemic region with high transmission of *Plasmodium falciparum* and *Plasmodium vivax*. Indigenous malaria caused by *P. vivax* was eliminated in Hainan in 2011, while imported vivax malaria remains. However, the geographical origin of *P. vivax* cases in Hainan remains unclear.

**Methods:**

Indigenous and imported *P. vivax* isolates (n = 45) were collected from Hainan Province, and the 6 kb mitochondrial genome was obtained. Nucleotide (*π*) and haplotype (*h*) diversity were estimated using DnaSP. The numbers of synonymous nucleotide substitutions per synonymous site (*d*_S_) and nonsynonymous nucleotide substitutions per nonsynonymous site (*d*_N_) were calculated using the SNAP program. Arlequin software was used to estimate the genetic diversity index and assess population differentiation. Bayesian phylogenetic analysis of *P. vivax* was performed using MrBayes. A haplotype network was generated using the NETWORK program.

**Results:**

In total, 983 complete mitochondrial genome sequences were collected, including 45 from this study and 938 publicly available from the NCBI. Thirty-three SNPs were identified, and 18 haplotypes were defined. The haplotype (0.834) and nucleotide (0.00061) diversity in the Hainan populations were higher than China’s Anhui and Guizhou population, and the majority of pairwise *F*_ST_ values in Hainan exceeded 0.25, suggesting strong differentiation among most populations except in Southeast Asia. Most Hainan haplotypes were connected to South/East Asian and China’s others haplotypes, but less connected with populations from China's Anhui and Guizhou provinces. Mitochondrial lineages of Hainan *P. vivax* belonged to clade 1 of four well-supported clades in a phylogenetic tree, most haplotypes of indigenous cases formed a subclade of clade 1, and the origin of seven imported cases (50%) could be inferred from the phylogenetic tree, but five imported cases (42.8%) could not be traced using the phylogenetic tree alone, necessitating epidemiological investigation.

**Conclusions:**

Indigenous cases in Hainan display high genetic (haplotype and nucleotide) diversity. Haplotype network analysis also revealed most haplotypes in Hainan were connected to the Southeast Asian populations and divergence to a cluster of China’s other populations. According to the mtDNA phylogenetic tree, some haplotypes were shared between geographic populations, and some haplotypes have formed lineages. Multiple tests are needed to further explore the origin and expansion of *P. vivax* populations.

**Supplementary Information:**

The online version contains supplementary material available at 10.1186/s12936-023-04520-7.

## Background

Malaria was one of the most important infectious diseases in China, where it had a wide geographical distribution [1]. Before the foundation of the People’s Republic of China (P.R. China) in 1949, it was estimated that 30 million malaria cases occurred yearly, and 70% of the counties were endemic for malaria [2–4]. Since then, organizations for malarial control and scientific research have been established, and large-scale surveys and anti-malaria campaigns have been carried out among regions with high transmission of malaria [5, 6]. To accelerate the process of malaria control and elimination, the National Malaria Control Programme (NMCP) from 2006 to 2015 and the Chinese Malaria Elimination Action Plan (2010–2020) (CMEAP) were sequentially formulated and issued [7, 8]. Subsequently, the scope of endemic areas was greatly reduced, and the number of cities and counties under control gradually increased, especially in the border counties of Yunnan and in mountainous counties of the central and southern parts of Hainan. Hainan and Yunnan were the provinces with the most serious endemic malaria transmission in the P.R. China before launching the NMCP, and their progress in elimination has had a significant impact on the overall prevalence of malaria in the country [9]. By 2009, the number of malaria patients dropped to approximately 14,000; the incidence in more than 95% of the counties dropped to below 1 per 10,000 [10].

Hainan Island accounts for most of the land in Hainan Province, which is the southernmost province in China. The tropical climate and environment of Hainan Island are within the suitable range for the breeding of *Anopheles dirus* and *Anopheles minimus,* and the characteristics of forest goers also facilitate malaria transmission [11]. Hainan Island was historically endemic for *Plasmodium vivax* and *Plasmodium falciparum* [12]. Geographically, malaria cases in Hainan Province were mainly distributed in patches in the southwest region of the island, while fewer cases were sparsely distributed in the northeast area [9]. A total of 12,225 (51.3%) *P. vivax* malaria cases, which accounted for nearly 10% of the cases nationwide, were reported from 2004 to 2012, and the reported *P. vivax* malaria cases in Hainan Island were mainly distributed in the southwestern counties of Qiongzhong (14.9%), Dongfang (13.8%), Baisha (12.4%), Ledong (11.9%) and Wanning (10.2%). After the implementation of the NMCP and CMEAP, the geographic range of locally transmitted malaria narrowed dramatically in Hainan, and the last indigenous case was *Plasmodium malariae* in 2015 and there was no indigenous case report for the first time in 2016 [11]. Additionally, imported *P. vivax* malaria cases cannot be neglected and have been reported in Hainan Province in the post-elimination stage.

Determining the geographic origin of an imported case poses a challenge when travel history is not recorded, and such gaps might have a long-term negative impact on the malaria surveillance system [13]. A new perspective offered by molecular genetics is employed to explore the geographic origin of infection, especially in the case of incomplete or missing epidemiological data. Genetic analysis is necessary for population genomic studies on *P. falciparum* and *P. vivax* cases [14–16]. The 6-kb mitochondrial (mt) genome of malaria parasites may have some advantages for epidemiological studies, although antigen-coding nuclear (*Pvmsp1, Pvcsp, Pvmsp3α* & *Pvmsp3β*) loci have revealed high genetic diversity before elimination in Hainan [17]. Additionally, the evolutionary history of *P. vivax* from other areas of China or other countries has recently been addressed [18–22], but the geographical origin of the *P. vivax* population in Hainan is not clear. In an attempt to answer this question, an analysis on population genetic of *P. vivax* isolates from Hainan Island was performed.

## Methods

### Sample collection

A total of 47 *P. vivax* parasite samples were previously collected in Hainan, and the source was verified by epidemiologic case investigation. All samples were stored in a refrigerator at -80°before usage. Two millilitre samples of whole blood were collected before treatment from patients who had symptoms of malaria from 2009 to 2020. All the samples were transported to Hainan Provincial Center for Disease Control and Prevention (Hi CDC) for further confirmation by microscope examination and PCR targeting the DNA of the *P. vivax* multicopy 18S ribosomal RNA gene [23]. These samples were treated with EDTA and then stored at − 20 °C until DNA extraction.

### DNA extraction, PCR amplification and DNA sequencing

DNA samples that were determined to be positive for *P. vivax* were used for subsequent amplification. The DNA was dissolved in TE buffer (10 mM Tris–HCl, pH 8.0, 0.1 M EDTA) and stored at − 20 °C until use. The whole mt DNA sequences (approx. 6 Kb) of the *P. vivax* isolates from Hainan were amplified by PCR and sequencing using oligonucleotide primers as previously described, with minor modifications [13].

Long-range, high-fidelity PCR amplification was performed using PrimeSTAR GXL DNA polymerase (Takara, Beijing, China), which has efficient 3ʹ → 5ʹ exonuclease proofreading activity. PCRs of 100 μl contained DNA template, each oligonucleotide primer at 0.2 μM, 1 × GXL PCR Buffer, 200 μM deoxynucleosides (dNTPs), and 5 units of polymerase mix. PCR was performed at 98 °C for 30 s, followed by 40 cycles of 98 °C for 10 s, 55 °C for 15 s, and 68 °C for 40 s. A final extension was performed at 68 °C for 3 min. PCR products were purified and sequenced by an ABI 3730XL DNA Analyzer at Guangzhou Tian Yihui Gene Technology Co., Ltd. All the PCR-amplified fragments were sequenced in both the forwards and reverse directions (6 × coverage), and two fragments of the complete *mt* genome in *P. vivax* were sequenced with 13 pairs of primers (Additional file 1).

The *mt* genome sequences were assembled, aligned, and annotated using the Geneious 11.0 program. DNA alignment of the whole mtDNA sequences of the *P. vivax* isolates was performed by Clustal W. The single complete mt sequences without repeat sequences were deposited in GenBank (OP250985-OP251004, OP320684-OP320708).

### Calculation of nucleotide diversity and haplotype diversity and assessment of population differentiation

For calculation and further analysis, sequences obtained in this study and 938 sequences publicly available from the NCBI (Mu et al. deposited 176 sequences [GenBank:AY791517.1-AY791692.1] [24]. Mu et al. deposited 106 sequences [GenBank: AY598035.1-AY598140.1] [24]. Iwagami et al. deposited 11 sequences [GenBank:AB550270.1-AB550280.1] [21]. Culleton et al. deposited 40 sequences [GenBank:JN788737.1-JN788776.1] [20]. Cox-Singh et al. [16] deposited 3 sequences [GenBank: DQ396547.1-DQ396549.1]. Taylor deposited 309 sequences [GenBank: KC330370-KC330678] [25]. Miao et al*.* deposited 99 sequences [GenBank: JQ240331-JQ240429] [18]. Rodrigues et al. deposited 67 sequences [GenBank: KF668361-KF668442] in 2014 and 127 sequences [GenBank: KY923298-KY923424] in 2018.) [13, 22]. Except for 17 sequences (KF668365, KF668366, KF668386, KF668394, KF668395, KF668397, KF668398, KF668405, KF668406, KF668409, KF668414, KF668415, KF668417, KF668419, KF668420, and KF668422) with an unknown origin of sampling, all sequence data had been previously published.

Nucleotide diversity (π) and haplotype diversity (*h*) were estimated using DnaSP version 5.6. The number of synonymous nucleotide substitutions per synonymous site (*d*_S_) and number of nonsynonymous nucleotide substitutions per nonsynonymous site (*d*_N_) were calculated using the SNAP program (http://www.hiv.lanl.gov/content/sequence/SNAP/SNAP.html) [18].

Arlequin 3.5 software was used to estimate genetic diversity indices and to assess population differentiation [26]. Pairwise comparisons of *F*_ST_ and *Φ*_ST_ values were carried out by permutation analyses using 1,000 permutations with an assumption of no difference between populations. The *P value* was calculated as the proportion of permutations resulting in an *F*_ST_ or *Φ*_ST_ value higher than or equal to the observed value. Analysis of molecular variance (AMOVA) was used to evaluate the extent to which sequence variation was partitioned among populations and geographical areas.

### Phylogenetic analysis and haplotype network construction

Bayesian phylogenetic analysis was carried out for *P. vivax* using MrBayes version 3.2.1 with two runs of four chains each [27], three heated and one cold, for 5 million generations. The phylogenetic tree was drawn using Dendroscope, and minor manual edits were performed for aesthetic purposes. Only unique haplotypes were included in the Bayesian phylogenetic analysis of GenBank-derived sequences; when identical haplotypes came from different regions, the geographic origin of one of them was randomly assigned to the haplotype. However, all imported malaria samples sequenced in this study are represented in the tree shown, even when two or more of them had identical haplotypes.

Median-joining phylogenies were generated using Network version 4.6 (Fluxus Technologies, http://www.fluxu-engeneering.com) with the default parameters and transversions weighted twice as high as transitions [28]. This analysis aimed to reconstruct global haplotype networks of the entire sets of *P. vivax* mt genomes, and the same colour code described above was used to show the geographic origins of the samples.

## Results

### Genetic variation in *P. vivax* mitochondrial genomes on Hainan Island

45 complete mt genomes from 47 *P. vivax* samples were obtained, excluding 2 cases because of failed amplification. Alignment of 45 complete mt genomes revealed 22 SNPs, including 12 transversions and 10 transitions, and 11 indels. Twelve of 22 SNPs were located in three coding regions (COX3, COX1 and CYTB). The 22 SNPs and 11 indels defined a total of 19 mt genome haplotypes, including 8 haplotypes in indigenous cases, 9 haplotypes in imported cases and 2 shared haplotype (Additional file 2).

Before comparing the haplotype and nucleotide diversities of *P. vivax* in Hainan with those worldwide, the complete mt genomes of indigenous cases were aligned, and the sequence of imported cases was designated to the responding geographic origin of infection from the seven geographical locations. The haplotypes diversity identified in Hainan populations (0.834) was moderate, higher than China's Anhui population (0.584) and China's Guizhou population (0.734), but lower than China's regions (0.875). The nucleotide diversity in the Hainan population was 0.00061 ± 0.00004, which was higher than that in China’s Guizhou and Anhui populations and nearly higher than that in China’s other regions and South and West Asia. Furthermore, the haplotype diversity in China (0.918) was also moderate, lower than that in Southeast Asia and South and West Asia (0.972–0.945) and higher than in Africa, Latin-America, Oceania and Korea (0.801–0.899) (Table 1).

**Table 1:** Summary of molecular diversity for all sampled *P. vivax* populations^a^

Geographic origin	Sample size	Date of collection	No. haplotypes	No. unique haplotypes	*S*	*h*	*π*	*k*	*d*_ S_	*d* _N_
China	130		54	50	30	0.918 ± 0.014	0.00080 ± 0.00004	4.667	0.9783	1.1472
Hainan	31^b^	2009–2011	11	9	14	0.834 ± 0.048	0.00061 ± 0.00004	3.566	0.3109	0.3827
Anhui	36	2004	11	8	14	0.584 ± 0.093	0.00020 ± 0.00006	1.211	0.0009	0.0003
Guizhou	29	2005	8	4	9	0.734 ± 0.060	0.00047 ± 0.00007	2.808	0.0009	0.0011
Other^c^	34		28	27	13	0.875 ± 0.036	0.00063 ± 0.00006	3.693	0.3214	0.3879
Korea	30	–	19	19	21	0.899 ± 0.037	0.00091 ± 0.00007	5.336	0.0012	0.0013
Southeast Asia^d^	168	–	127	120	105	0.972 ± 0.006	0.00096 ± 0.00006	5.546	0.2245	0.2587
South and West Asia^d^	134	–	106	95	93	0.945 ± 0.015	0.00056 ± 0.00004	3.279	0.0623	0.0745
Africa	94	–	63	55	80	0.801 ± 0.045	0.00042 ± 0.00005	2.445	0.2159	0.2159
Latin-America	298^*^	–	159	158	132	0.855 ± 0.020	0.00480 ± 0.00003	2.788	0.0014	0.0005
Oceania	129	–	82	79	56	0.866 ± 0.024	0.00044 ± 0.00004	2.572	0.0515	0.0585
Total	983	–	592	573	377	0.973 ± 0.0025	0.00086 ± n.d	4.973	0.1157	0.1392

^a^Shown are the number of segregating sites (*S*), haplotype diversity (*h* ± standard deviation), nucleotide diversity (*π* ± standard deviation), average pairwise difference among individuals (k ± total variance), the average numbers of nucleotide substitutions per nonsynonymous site (*d*_N_) and synonymous site (*d*_S_) for each grouping of samples, calculated based on the mitochondrial DNA sequences

^b^Sequences from Hainan indigenous cases were included from reference [24]

^c^Additional sequences from China were obtained from Guangxi, Yunnan and others from references [18, 24, 29] and an unknown isolate from Hainan

^d^Samples from Thailand and Vietnam were grouped within Southeast Asia, which also included Laos, Cambodia, Myanmar, Malaysia, Singapore, Indonesia, Brunei, the Philippines, and Timor-Leste; South and West Asia included Nepal, Bhutan, India, Pakistan, Bangladesh, Sri Lanka, the Maldives, Iran, Iraq, Azerbaijan, Georgia, Armenia, Turkey, Syria, Jordan, Israel, Palestine, Saudi Arabia, Bahrain, Qatar, Yemen, Oman, the United Arab Emirates, Kuwait, Lebanon, Cyprus, and Afghanistan; Oceania refers to Melanesia

^*^Excluding samples from unknown American origin

### Population differentiation and structure

*F*_ST_ (based only on haplotype frequency) and *Φ*_ST_ (based on genetic distance) were estimated to further determine population differentiation for each population by mtDNA sequences. The majority of pairwise *F*_ST_ values from Hainan were greater than 0.25 (ranging from 0.15 to 0.75), suggesting strong population differentiation among most populations except those from Southeast Asia, which showed moderate genetic differentiation (*F*_ST_ = 0.15, *P* < 0.05; *Φ*_ST_ = 0.04). Compared with Southeast Asia, Hainan was less distinct, with *F*_ST_ values of 0.1588 and *Φ*_ST_ values of 0.0466 in the world. In the Chinese *P. vivax* populations, which included Anhui, Guizhou and other populations, genetic differences between populations were strong (*F*_ST_ = 0.45–0.57, *Φ*_ST_ = 0.16–0.05) (Table 2). All studied populations were grouped into eight geographical groups consisting of Africa, America, South/West Asia, Southeast Asia, China, Korea, and Oceania and performed hierarchical AMOVA. The three covariance components (within populations, among populations/within groups, and among populations) explained 6.68%, 25.6 and 67.7% of the variance, respectively (Additional file 3). Thus, considerable variation was preserved at the population level.

**Table 2:** Pairwise *F*_ST_ (below diagonal) and *Φ*_ST_ (above diagonal) values of worldwide *P. vivax* populations

	China	Hainan, China	Anhui, China	Guizhou, China	Otherc	Korea	Southeast Asia	South and West Asia	Africa	South America	Oceania
China (CN)	–	–	–	–	–	0.0462	0.0233	0.0282	0.0428	0.0615	0.0443
Hainan (HN)	–	–	0.166	0.1811	0.0581	0.0779	0.0466	0.0568	0.0723	0.0910	0.0736
Anhui (AH)	–	0.5728	–	0.1708	0.1187	0.1356	0.1033	0.1075	0.1241	0.1393	0.1242
Guizhou (GZ)	–	0.4766	0.2639	–	0.1321	0.1494	0.1151	0.1194	0.1363	0.1512	0.1362
Otherc (OT)	–	0.4553	0.5884	0.5540	–	0.0300	0.0096	0.0120	0.0270	0.0469	0.0287
Korea (KO)	0.6655	0.7548	0.8227	0.8000	0.4298	–	0.0249	0.0272	0.0424	0.0620	0.0439
Southeast Asia (SE)	0.1337	0.1588	0.2594	0.2352	0.1712	0.3673	–	0.0068	0.0221	0.0412	0.0238
Southwest Asia (SW)	0.4763	0.5005	0.5701	0.5506	0.2819	0.1290	0.1989	–	0.0194	0.0435	0.0258
Africa (AF)	0.5785	0.6162	0.6788	0.6592	0.3437	0.0941	0.3010	0.0353	–	0.0581	0.0406
Latin-American(AM)	0.3102	0.3227	0.4123	0.3936	0.2610	0.2882	0.0794	0.0988	0.2051	–	0.0593
Oceania (OC)	0.6259	0.7046	0.7618	0.7481	0.2924	0.4481	0.4184	0.3246	0.2982	0.3964	–

Pairwise *F*_ST_ was calculated by using conventional F-statistics (haplotype frequencies only), and *Φ*_ST_ was calculated by using a project distance matrix. The *F*_ST_ value was defined as follows: below 0.05 indicating a small degree of genetic differentiation among the populations; from 0.05 to 0.15 indicating a moderate degree of genetic differentiation; from 0.15 to 0.25 indicating great genetic differentiation; and above 0.25 indicating much great genetic differentiation [30]

### Phylogeography

The obtained 45 sequences referred to 30 indigenous cases, 14 imported cases and one unclassified case. Sequentially, 30 indigenous cases collected from 2009 to 2011, 14 imported cases from 2011 to 2020 and 1 unclassified case in 2010. Analysis of *P. vivax* mt genomes from 45 newly collected Hainan samples as well as 938 previously described isolates from 10 populations revealed a total of 592 haplotypes.

**Fig. 1:**
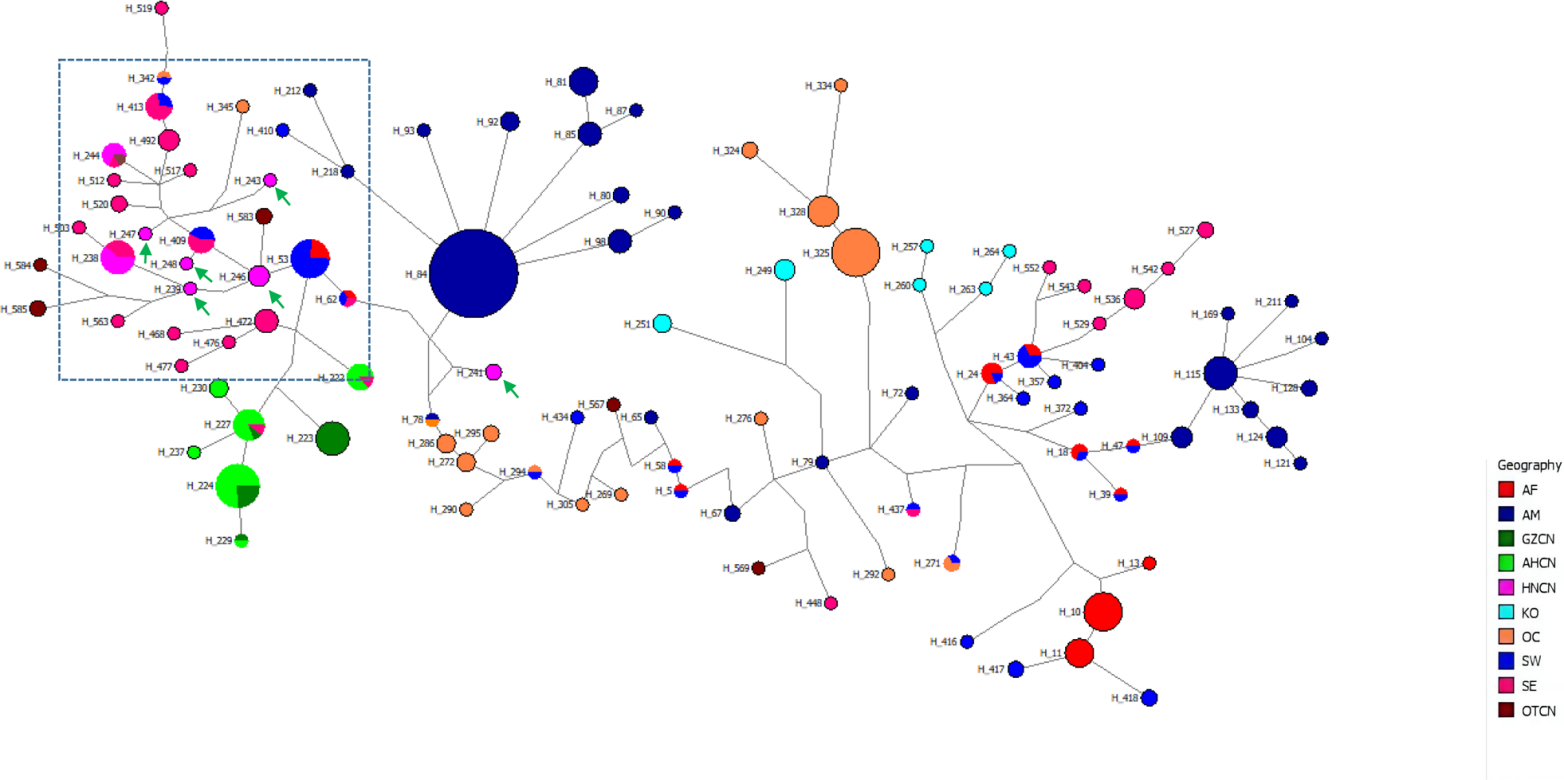
Median-joining network of a worldwide collection of *P. vivax* mitochondrial genome haplotypes, including 45 newly collected Hainan samples as well as 938 samples previously described in other studies. Circles represent haplotypes, and their sizes are proportional to haplotype frequencies. Colours indicate the regions of origin of the samples, as in Fig. 1. Dark blue = the Americas; brown = other regions in China (including Guangxi, Yunnan and unknown); dark green = Guizhou in China; light green = Anhui in China; cherry red = Southeast Asia; light blue = South Asia; pink = Hainan in China; orange = Oceania; sky-blue = Korea; red = Africa. Each line connecting the circles represents a mutational step. The dashed boxes represents indigenouse cases in Hainan, and the green arrows indicate haplotypes in Hainan

To characterize the frequencies and relationships of different haplotypes, a minimum spanning tree was constructed. This haplotype network clearly showed geographical clustering of the haplotypes (Fig. 1). For the Hainan populations, most haplotypes were connected to the South/East Asian populations, but the haplotypes were less connected with populations from China's Anhui and Guizhou provinces, which formed a local network in central China. Furthermore, two clusters were formed in China’s other populations (some from Guangxi Province in southern China) and connected with a haplotype from Hainan. Although the haplotypes segregated according to geographical location, many were shared between locations. Two haplotypes from indigenous cases have shared with other regions, one shared with southeast Asia and China’s other, the other with southeast Asia. Further, two haplotypes from imported cases also have shared, one with southeast Asia and Southwest Asia, the other with Southwest Asia. As to other haplotypes in other geographical location, the shared haplotypes have showed and listed in Additional 4. Interestingly, haplotypes from China’s Anhui and Guizhou provinces and from Hainan and other regions were connected to the South/West Asian populations, and two separate topological branches were shown in this study.

**Fig. 2:**
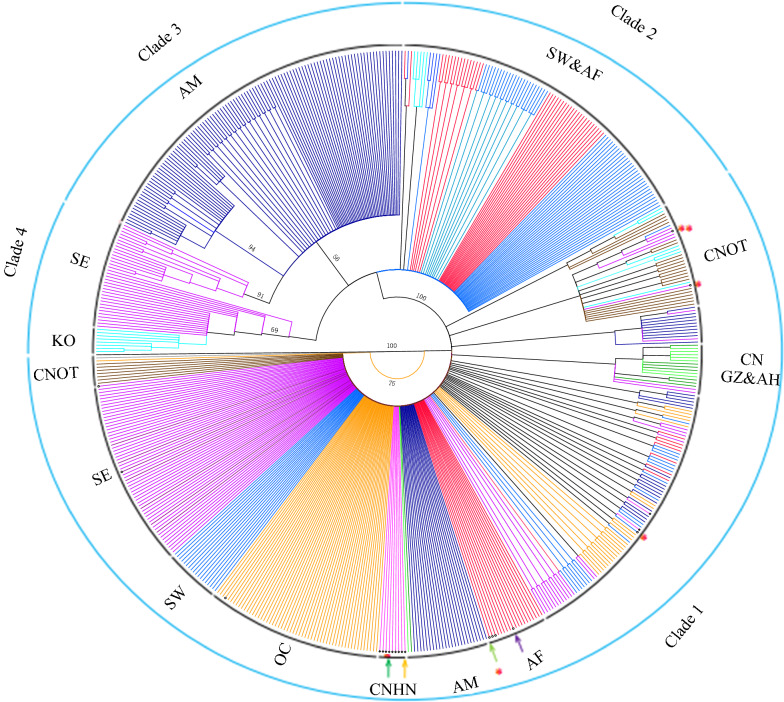
Bayesian phylogenetic analysis of unique haplotype sequences from the worldwide *P. vivax* mt genome collection, including haplotypes from imported malaria cases and indigenous cases in this study (imported malaria cases labelled with a circle and indigenous cases labelled with a diamond). The posterior probabilities of selected clades (clades with values > 50) are listed next to the corresponding branches. We labelled 16 of 17 well-supported clades with the abbreviation of their geographic origin. Only unique samples were included in the phylogenetic tree. The trees were drawn using Dendroscope version 3.4 software 72, and a colour code is applied to identify the geographic origins of parasites: AF (red), AM (dark blue), SW (light blue), SE (purple), OC (orange), CNHN (pink), CNGZ&AH (green), and CNOT (brown). Arrow indicates the shared haplotype in this study, deep green with SE and ot, Orange with SE, light green with SW and SE, Purple with AF and SW. * showed mismatch on the origin between in epidemical information with phylogenetic analysis

To further illustrate the relationships of the mt genome haplotypes, a maximum likelihood phylogenetic tree based on the complete genome sequences was constructed. Four well-supported clades (posterior probability > 0.5) comprised all haplotypes: clade 1 (80 identical haplotypes from OC, 53 from CN, 82 from SA, 38 from SW, 50 from AM, 26 from AF, and 6 from KO), clade 2 (56 haplotypes from SW, 38 from AF and 5 from KO), clade 3 (110 haplotypes from AM and 55 from SE), and clade 4 (19 haplotypes from SE and 8 from KO). Hainan’s mt lineages of *P. vivax* belonged to clade 1, which are most widely spread across the Bayesian phylogenetic tree (Fig. 2). Interestingly, most haplotypes of indigenous cases belong to a subclade of clade 1, and the origins of eight imported cases (57.1%, 8/14) were traced using the phylogenetic tree, consistent with results from travel histories. However, six imported cases (42.9%, 6/14) could not be traced only using the phylogenetic tree, necessitating epidemiological investigation. Additionally, an unknown sample lacking an informative travel history could be assigned to the Hainan lineage of geographic populations in the phylogenetic tree, and Chinese lineages of *P. vivax* belonged to clade 1 in the phylogenetic tree.

## Discussion

China officially achieved the elimination of malaria on June 30, 2021 [1], but new infections continue to be imported into China, mainly by migrants and travellers coming from areas with ongoing malaria transmission. Before the COVID-19 pandemic, there were approximately 3,000 imported cases every year, mainly from Africa and Southeast Asia [10]. Five *Plasmodium* species were reported, with *P. falciparum* being the most prevalent, followed by *P. vivax* [10]. These imported malaria cases were widely distributed in historically malaria-endemic provinces in China. Additionally, these cases were reported throughout the year, and June and July were the cumulative peak periods, especially in historically malaria-endemic areas [31]. Those imported cases have overlapped with past indigenous cases in transmission season which has unique characteristics with short incubation period and seasonal transmission thoughout the year [9]. Therefore, imported cases of *P. vivax* should be given greater attention, although indigenous cases have been eliminated in 2021.

According to the results of this study, indigenous cases in Hainan display high genetic diversity in terms of haplotype diversity and nucleotide diversity, as reported in *pvmsp1* and *pvcsp* [17]. The mt genome from analysis also revealed comparable, high-level genetic diversity among Hainan’s *P. vivax* populations. Consistent with high malaria endemicity in Myanmar, haplotype diversity was also high (0.85 ± 0.057) and comparable to that in other highly endemic areas of the world [24]. We thus speculate that the high diversity is related to the mechanism of transmission in the parasite population. Transmission intensity affects diversity and population structure have been showed by four continents [32]. This phenomenon in the *P. vivax* population was also found in tropical countries where *Anopheles dirus* and *Anopheles minimus* act as primary vectors. In the Greater Mekong Subregion, complex vector species relates to transmission intensity, and the latter further lead to high diversity in the parasite population. Historically, the Guizhou and Anhui populations were defined as temperate *P. vivax* malarial populations with characteristics of a long incubation period, seasonal transmission by *Anopheles sinensis* or *Anopheles anthropophagus* and a mechanism of adaptation to a cold climate [33]. However, Hainan’s population acted as tropical *P. vivax*, which is transmitted by *An. dirus* and *An. minimus* throughout the year. The haplotype diversity of Anhui’s and Guizhou’s populations in China was lower or similar to that of other populations in China.

In a finite population, a pattern of genetic isolation by geographic distance is generally expected [34], and this principle also applies to *P. vivax* populations, especially those on different continents [24, 35]. In this study, *F*_ST_ and *Φ*_ST_ statistics revealed significant differentiation between Hainan and other *P. vivax* populations in China. Interestingly, three major genotypes in China were also observed: Hainan, Guizhou and Anhui, and others. Generally, old (or ancestral) populations are more genetically diverged than young populations [21]. The genetic diversity of global *P. vivax* populations is thought to be the result of ancient hominid geographical expansion [29]. Genetic differentiation may be significant due to possible migration or ecological constraints [36]. In this context, the Hainan populations and Southeast Asian populations seemed to be old populations.

Most mtDNA haplotypes from Hainan population were unique but related, suggesting that they might be descendants from the same lineage(s). Haplotype network analysis suggested Southwest Asia as the root or origin of the parasite populations, which is also verified by research on *P. vivax* from temperate regions in East and Southeast Asia [18]. The location of Southwest Asia alike a center for globe population expansion, and every population have connection with Southwest Asia. The population of Southwest Asia cannot be neglected between African and American populations, although the present-day African and American populations may be the closest extant relatives of the African ancestor [20]. Haplotype network analysis also showed that samples collected in China formed two independent and divergent lineages: one was closely related to the Hainan sample and most Southeast Asian samples, whereas the other was clustered with Anhui and Guizhou temperate samples and some samples from Southeast Asia. Since clustering in the network is often affected by the methodologies used, the exact origin of Hainan’s vivax ancestor is still not clear. Additionally, all haplotypes from China belonged to clade 1, unlike the Korean lineages, which formed two divergent lineages, one closely related to the Oceania samples and another directly diverged from African samples. These results are not consistent with the notion that the genealogical origin of Korean lineages is related to southern China [21]. Furthermore, the findings suggest that two kinds of relapsing hypnozoites, which present a long or short incubation period [37], may represent two unique lineages in China, with adaptation to the local climate for transmission with or without interruption in winter.

In the mtDNA phylogenetic tree, some haplotypes were found to be shared by different geographic populations, which made it difficult to trace the origins of geographic populations with missing travel histories, although most haplotypes formed lineages undergoing population expansion. The result from origin tracing is consistent with imported malaria cases in the USA, which show some closely shared haplotypes and chaos in lineages from different geographic populations [13]. Thus, more robust data from whole genome from nuclear genes or from both nuclear and mt genomes, especially from samples in Southwest Asia and Southeast Asia, are needed to corroborate this conclusion.

## Conclusion

The present study addressed extant *P. viva*x population structure by focusing on Hainan populations in China. Analysis of the complete mt genomes from 45 clinical samples confirmed that *P. vivax* displays extensive genetic diversity and that natural populations are clearly structured. Most of indigenous case have formed a unique lineages and two haplotypes were shared with Southeast Asia. While most mtDNA haplotypes from Hainan were related to Southeast Asian haplotypes, they were different from those collected in the centre of China, suggesting that Southwest Asia is the root and that local population subdivision occurs. Multiple tests are needed to further explore the origin and expansion of *P. vivax* populations.

## Supplementary Information

**Additional file 1: **Primer sequences. Primers used for amplifying and sequencing the mitochondrial genome of *Plasmodium vivax*.

**Additional file 2: **Alignment of the complete mt genomes identified 33 SNPs from Hainan.

**Additional file 3: **The calculation for covariance components within population, among population/within group, and among populations.

**Additional file 4: **Haplotypes in all geographical location.

## Data Availability

All data supporting the conclusions of this study are included in the article.

## Abbreviations

NMCP: National Malaria Control Programme
CMEAP: Chinese Malaria Elimination Action Plan (2010–2020)
CNHN: Hainan, China
CNAH: Anhui, China
CNGZ: Guizhou, China
CNOT: Other, China
KO: Korea
SE: Southeast Asia
SW: Southwest Asia
AF: Africa
AM: America
OC: Oceania
