# Supplementary material for: Geographical origin of Plasmodium vivax in the Hainan Island, China: insights from mitochondrial genome
Source: Malar J. 2023 Mar 8;22:84. doi: 10.1186/s12936-023-04520-7 (PMC9993381; doi:10.1186/s12936-023-04520-7)
Supplement: Supplementary file 1 — Additional file 1: Primer sequences. Primers used for amplifying and sequencing the mitochondrial genome of Plasmodium vivax. [file 12936_2023_4520_MOESM1_ESM.docx]

**Additional file 1: Primer sequences.** Primers used for amplifying and sequencing the mitochondrial genome of *Plasmodium vivax*.

| **Primer** | **Amplification /Sequencing** | **Sequence (5'-3')** |
| --- | --- | --- |
| Pvmit 1F | amplification/sequencing | CGCTGACTTCCTGGCTAAAC |
| Pvmit 1R | amplification/sequencing | GTCAGGCGTTAAAAGCGTTC |
| Pvmit 2F | amplification/sequencing | TTGTACACACCGCTCGTCAC |
| Pvmit 2R | amplification/sequencing | CCGAACCTTGGACTCTTGAA |
| mtDNA1_F | sequencing | ACGCTGACTTCCTGGCTAAA |
| mtDNA1_R | sequencing | GGATGAAACCTTCCTGATCG |
| mtDNA2_F | sequencing | CACGAGTCGATCAGGAAGGT |
| mtDNA2_R | sequencing | GTGTTGGCTGGGCATTAGTC |
| mtDNA3_F | sequencing | GACTAATGCCCAGCCAACAC |
| mtDNA3_R | sequencing | GCTATCAAATGGCGAGAAGG |
| mtDNA4_F | sequencing | CCTTCTCGCCATTTGATAGC |
| mtDNA4_R | sequencing | GCATCATGTATGACAGCATGTTT |
| mtDNA5_F | sequencing | TGCTGTCATACATGATGCACTT |
| mtDNA5_R | sequencing | CAAGGCAACAATACACGCTAA |
| mtDNA6_F | sequencing | AGCGTGTATTGTTGCCTTGT |
| mtDNA6_R | sequencing | CATCCATGTCAGGCGTTAAA |
| mtDNA7_F | sequencing | TTGTACACACCGCTCGTCAC |
| mtDNA7_R | sequencing | AACTACCAAATAAAAATGAAAACCA |
| mtDNA8_F | sequencing | CAAATTGCAATCATAAAACTTTAGGTC |
| mtDNA8_R | sequencing | CTAGCAATACCAGATACTAAAAGACCA |
| mtDNA9_F | sequencing | TCATTGTTGGTCTTTTAGTATCTGG |
| mtDNA9_R | sequencing | CCAATTAAATATTTTTGTTCCAGTAGG |
| mtDNA10_F | sequencing | CCTACTGGAACAAAAATATTTAATTGG |
| mtDNA10_R | sequencing | TTTAATGGGCATGGGTAATTT |
| mtDNA11_F | sequencing | AAATTACCCATGCCCATTAAA |
| mtDNA11_R | sequencing | CCCTAAAGGATTTGTGCTACC |
| mtDNA12_F | sequencing | TGGTAGCACAAATCCTTTAGGG |
| mtDNA12_R | sequencing | AAATGTTTGCTTGGGAGCTG |
| mtDNA13_F | sequencing | ACAGCTCCCAAGCAAACATT |
| mtDNA13_R | sequencing | GACCGAACCTTGGACTCTTG |

PCR reactions were performed in 50 μL total reaction volume containing 3 μL DNA template,0.2mM each oligonucleotide primer, 10 ×PrimeSTAR GXL PCR Buffer, 2.5 mM each deoxynucleoside (dNTP), and 2.5 units PrimeSTAR GXL DNA polymerase. PCR was performed at 94°C for 1 minute followed by 30 cycles at 98°C for 10 seconds, 55°C for 15 seconds, and 68°C for 40 seconds. A final extension was done at 68°C for 3 minutes. The amplified PCR products were resolved on 1.5% agarose gel, and the sizes of the PCR products were determined using a D2000 DNA ladder (Tiangen, China).
