# Supplementary figures and images for: Geographical origin of Plasmodium vivax in the Hainan Island, China: insights from mitochondrial genome
Source: Malar J. 2023 Mar 8;22:84. doi: 10.1186/s12936-023-04520-7 (PMC9993381; doi:10.1186/s12936-023-04520-7)

Additional file 2 Alignment of the complete mt genomes identified 33 SNPs from Hainan


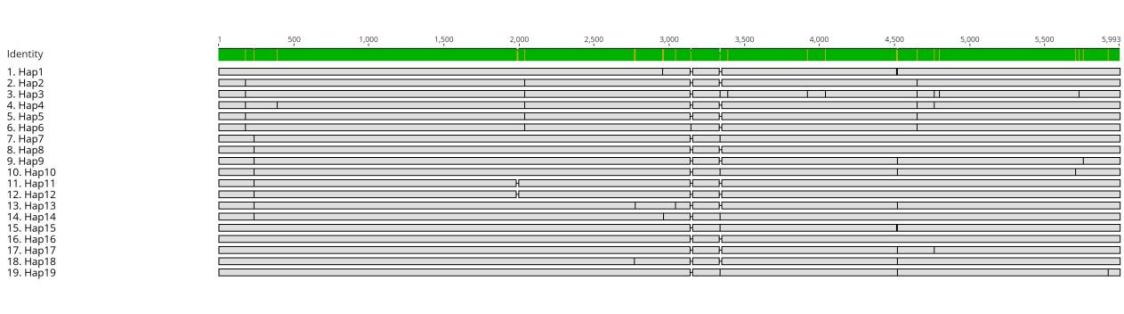

Supplement: Supplementary file 2 — Additional file 2: Alignment of the complete mt genomes identified 33 SNPs from Hainan. [file 12936_2023_4520_MOESM2_ESM.docx]
