# Supplementary material for: Geographical origin of Plasmodium vivax in the Hainan Island, China: insights from mitochondrial genome
Source: Malar J. 2023 Mar 8;22:84. doi: 10.1186/s12936-023-04520-7 (PMC9993381; doi:10.1186/s12936-023-04520-7)
Supplement: Supplementary file 3 — Additional file 3: The calculation for covariance components within population, among population/within group, and among populations. [file 12936_2023_4520_MOESM3_ESM.docx]

# Additional file 3: The calculation for covariance components within population, among population/within group, and among populations

| Source of variation | d.f. | Sum of squares | Variance components | Percentage of variation |
| --- | --- | --- | --- | --- |
| Among groups | 6 | 9133.198 | 2.52908 | 6.68 |
| Among populations within | 3 | 1019.792 | 9.69248 | 25.60 |
| Groups within populations | 973 | 24953.245 | 25.64568 | 67.73 |
| Total | 982 | 35106.235 | 37.86724 | 100 |

# Fixation Indices

# *F*_SC_ *: 0.27428*

# *F*_ST_ *: 0.32275*

# *F*_CT_ *: 0.06679*
