# Supplementary material for: Geographical origin of Plasmodium vivax in the Hainan Island, China: insights from mitochondrial genome
Source: Malar J. 2023 Mar 8;22:84. doi: 10.1186/s12936-023-04520-7 (PMC9993381; doi:10.1186/s12936-023-04520-7)
Supplement: Supplementary file 4 — Additional file 4: Haplotypes in all geographical location. [file 12936_2023_4520_MOESM4_ESM.docx]

Additional file 4: Haplotypes in all geographical location.

Hap_1: 1 [AY791525.1af]

Hap_2: 1 [AY791524.1af]

Hap_3: 1 [AY791523.1af]

Hap_4: 1 [AY791522.1af]

Hap_5: 1 [AY791521.1af]

Hap_6: 1 [AY791520.1af]

Hap_7: 1 [AY791519.1af]

Hap_8: 1 [AY791518.1af]

Hap_9: 1 [AY791517.1af]

Hap_10: 16 [JN788766.1af JN788763.1af JN788761.1af JN788760.1af JN788758.1af JN788756.1af JN788755.1af JN788753.1af JN788751.1af JN788750.1af JN788746.1af JN788744.1af JN788743.1af JN788741.1af JN788739.1af JN788738.1af]

Hap_11: 9 [JN788765.1af JN788762.1af JN788759.1af JN788757.1af JN788752.1af JN788747.1af JN788745.1af JN788742.1af JN788737.1af]

Hap_12: 1 [JN788764.1af]

Hap_13: 2 [JN788754.1af JN788740.1af]

Hap_14: 1 [JN788749.1af]

Hap_15: 1 [JN788748.1af]

Hap_16: 1 [KC330478.1af]

Hap_17: 1 [KC330477.1af]

Hap_18: 3 [KC330476.1af KC330451.1af KC330436.1SW]

Hap_19: 1 [KC330475.1af]

Hap_20: 1 [KC330474.1af]

Hap_21: 1 [KC330473.1af]

Hap_22: 1 [KC330472.1af]

Hap_23: 1 [KC330471.1af]

Hap_24: 5 [KC330470.1af KC330469.1af KC330468.1af KC330467.1af KC330392.1SW]

Hap_25: 1 [KC330466.1af]

Hap_26: 1 [KC330465.1af]

Hap_27: 1 [KC330464.1af]

Hap_28: 1 [KC330463.1af]

Hap_29: 1 [KC330462.1af]

Hap_30: 1 [KC330461.1af]

Hap_31: 1 [KC330460.1af]

Hap_32: 1 [KC330459.1af]

Hap_33: 1 [KC330458.1af]

Hap_34: 1 [KC330457.1af]

Hap_35: 1 [KC330456.1af]

Hap_36: 1 [KC330455.1af]

Hap_37: 1 [KC330454.1af]

Hap_38: 1 [KC330453.1af]

Hap_39: 2 [KC330452.1af KC330412.1SW]

Hap_40: 1 [KC330450.1af]

Hap_41: 1 [KC330449.1af]

Hap_42: 1 [KC330448.1af]

Hap_43: 6 [KC330447.1af KC330446.1af KC330434.1SW KC330428.1SW KC330389.1SW KC330388.1SW]

Hap_44: 1 [KC330445.1af]

Hap_45: 1 [KC330444.1af]

Hap_46: 1 [KC330443.1af]

Hap_47: 2 [KC330442.1af KC330403.1SW]

Hap_48: 1 [KC330441.1af]

Hap_49: 1 [KC330440.1af]

Hap_50: 1 [KC330480.1af]

Hap_51: 1 [KC330479.1af]

Hap_52: 1 [KF668362.1af]

Hap_53: 16 [KF668380.1af KF668411.1af KF668423.1af op320693.1AF KF668361.1SW KF668367.1SW KF668370.1SW KF668381.1SW KF668382.1SW KF668412.1SW KF668421.1SW KF668425.1SW KF668426.1SW KF668429.1SW AY598120.1SW KF668399.1SW]

Hap_54: 1 [KF668389.1af]

Hap_55: 1 [KF668392.1af]

Hap_56: 1 [AY791528.1af]

Hap_57: 1 [AY791527.1af]

Hap_58: 1 [AY791526.1af]

Hap_59: 1 [KF668401.1af]

Hap_60: 1 [op250990.1af]

Hap_61: 1 [op250991.1AF]

Hap_62: 3 [op320695.1AF op320694.1SW op250988.1SE]

Hap_63: 1 [AY791550.1AM]

Hap_64: 1 [AY791549.1AM]

Hap_65: 2 [AY791548.1AM AY791540.1AM]

Hap_66: 1 [AY791547.1AM]

Hap_67: 1 [AY791546.1AM]

Hap_68: 1 [AY791545.1AM]

Hap_69: 1 [AY791544.1AM]

Hap_70: 2 [AY791543.1AM AY791542.1AM]

Hap_71: 1 [AY791539.1AM]

Hap_72: 2 [AY791538.1AM AY791534.1AM]

Hap_73: 1 [AY791537.1AM]

Hap_74: 1 [AY791536.1AM]

Hap_75: 1 [AY791535.1AM]

Hap_76: 1 [AY791533.1AM]

Hap_77: 1 [AY791532.1AM]

Hap_78: 2 [AY791531.1AM AY791673.1Oc]

Hap_79: 2 [AY791530.1AM AY791529.1AM]

Hap_80: 3 [AY598140.1AM KF668431.1AM KF668435.1AM]

Hap_81: 9 [KY923372.1AM KY923369.1AM KY923366.1AM KY923363.1AM KY923361.1AM KY923360.1AM KY923359.1AM KY923357.1AM KY923354.1AM]

Hap_82: 1 [KY923371.1AM]

Hap_83: 1 [KY923370.1AM]

Hap_84: 80 [KY923368.1AM KY923367.1AM KY923350.1AM KY923349.1AM KY923348.1AM KY923347.1AM KY923346.1AM KY923345.1AM KY923344.1AM KY923343.1AM AY598097.1AM AY598095.1AM AY598089.1AM AY598088.1AM AY598087.1AM AY598086.1AM AY598083.1AM AY598082.1AM AY598081.1AM AY598080.1AM AY598079.1AM AY598077.1AM AY598075.1AM AY598074.1AM KY923341.1AM KY923340.1AM KY923339.1AM KY923338.1AM KY923337.1AM KY923336.1AM KY923335.1AM KY923333.1AM KY923331.1AM KY923330.1AM KY923329.1AM KY923328.1AM KY923327.1AM KY923326.1AM KY923325.1AM KY923324.1AM KY923322.1AM KY923321.1AM KY923320.1AM KY923319.1AM KY923314.1AM KY923311.1AM KY923310.1AM KY923309.1AM KY923308.1AM KY923307.1AM KY923304.1AM KY923303.1AM KY923301.1AM KY923299.1AM KF668363.1AM KF668364.1AM KF668371.1AM KF668372.1AM KF668374.1AM KF668376.1AM KF668377.1AM KF668378.1AM KF668383.1AM KF668393.1AM KF668408.1AM KF668430.1AM KF668432.1AM KF668433.1AM KF668434.1AM KF668436.1AM KF668437.1AM KF668439.1AM KF668440.1AM KF668442.1AM KF668369.1AM KF668375.1AM KF668384.1AM KF668388.1AM KF668416.1AM KF668418.1AM]

Hap_85: 6 [KY923365.1AM KY923362.1AM KY923358.1AM KY923356.1AM KY923353.1AM KY923351.1AM]

Hap_86: 1 [KY923364.1AM]

Hap_87: 2 [KY923355.1AM KY923352.1AM]

Hap_88: 1 [KY923342.1AM]

Hap_89: 1 [AY598096.1AM]

Hap_90: 2 [AY598094.1AM AY598090.1AM]

Hap_91: 1 [AY598093.1AM]

Hap_92: 4 [AY598092.1AM AY598091.1AM AY598085.1AM AY598084.1AM]

Hap_93: 2 [AY598078.1AM AY598076.1AM]

Hap_94: 1 [KY923334.1AM]

Hap_95: 1 [KY923332.1AM]

Hap_96: 1 [KY923323.1AM]

Hap_97: 1 [KY923318.1AM]

Hap_98: 6 [KY923317.1AM KY923315.1AM KY923313.1AM KY923305.1AM KY923300.1AM KY923298.1AM]

Hap_99: 1 [KY923316.1AM]

Hap_100: 1 [KY923312.1AM]

Hap_101: 1 [KY923306.1AM]

Hap_102: 1 [KY923302.1AM]

Hap_103: 1 [KC330385.1AM]

Hap_104: 2 [KC330384.1AM KC330580.1AM]

Hap_105: 1 [KC330383.1AM]

Hap_106: 1 [KC330382.1AM]

Hap_107: 1 [KC330381.1AM]

Hap_108: 1 [KC330380.1AM]

Hap_109: 5 [KC330379.1AM KC330668.1AM KC330655.1AM KC330654.1AM KC330572.1AM]

Hap_110: 1 [KC330378.1AM]

Hap_111: 1 [KC330377.1AM]

Hap_112: 1 [KC330376.1AM]

Hap_113: 1 [KC330375.1AM]

Hap_114: 1 [KC330374.1AM]

Hap_115: 13 [KC330373.1AM KC330372.1AM KC330636.1AM KC330605.1AM KC330604.1AM KC330603.1AM KC330602.1AM KC330601.1AM KC330600.1AM KC330599.1AM KC330563.1AM KC330562.1AM KC330558.1AM]

Hap_116: 1 [KC330371.1AM]

Hap_117: 1 [KC330370.1AM]

Hap_118: 1 [KC330678.1AM]

Hap_119: 1 [KC330677.1AM]

Hap_120: 1 [KC330676.1AM]

Hap_121: 2 [KC330675.1AM KC330593.1AM]

Hap_122: 1 [KC330674.1AM]

Hap_123: 1 [KC330673.1AM]

Hap_124: 5 [KC330672.1AM KC330624.1AM KC330623.1AM KC330588.1AM KC330576.1AM]

Hap_125: 1 [KC330671.1AM]

Hap_126: 1 [KC330670.1AM]

Hap_127: 1 [KC330669.1AM]

Hap_128: 3 [KC330667.1AM KC330653.1AM KC330639.1AM]

Hap_129: 1 [KC330666.1AM]

Hap_130: 1 [KC330665.1AM]

Hap_131: 1 [KC330664.1AM]

Hap_132: 1 [KC330663.1AM]

Hap_133: 3 [KC330662.1AM KC330648.1AM KC330647.1AM]

Hap_134: 1 [KC330661.1AM]

Hap_135: 1 [KC330660.1AM]

Hap_136: 1 [KC330659.1AM]

Hap_137: 1 [KC330658.1AM]

Hap_138: 1 [KC330657.1AM]

Hap_139: 1 [KC330656.1AM]

Hap_140: 1 [KC330652.1AM]

Hap_141: 1 [KC330651.1AM]

Hap_142: 1 [KC330650.1AM]

Hap_143: 1 [KC330649.1AM]

Hap_144: 1 [KC330646.1AM]

Hap_145: 1 [KC330645.1AM]

Hap_146: 1 [KC330644.1AM]

Hap_147: 1 [KC330643.1AM]

Hap_148: 1 [KC330642.1AM]

Hap_149: 1 [KC330641.1AM]

Hap_150: 1 [KC330640.1AM]

Hap_151: 1 [KC330638.1AM]

Hap_152: 1 [KC330637.1AM]

Hap_153: 1 [KC330635.1AM]

Hap_154: 1 [KC330634.1AM]

Hap_155: 1 [KC330633.1AM]

Hap_156: 1 [KC330632.1AM]

Hap_157: 1 [KC330631.1AM]

Hap_158: 1 [KC330630.1AM]

Hap_159: 1 [KC330629.1AM]

Hap_160: 1 [KC330628.1AM]

Hap_161: 1 [KC330627.1AM]

Hap_162: 1 [KC330626.1AM]

Hap_163: 1 [KC330625.1AM]

Hap_164: 1 [KC330622.1AM]

Hap_165: 1 [KC330621.1AM]

Hap_166: 1 [KC330620.1AM]

Hap_167: 1 [KC330619.1AM]

Hap_168: 1 [KC330618.1AM]

Hap_169: 2 [KC330617.1AM KC330561.1AM]

Hap_170: 1 [KC330616.1AM]

Hap_171: 1 [KC330615.1AM]

Hap_172: 1 [KC330614.1AM]

Hap_173: 1 [KC330613.1AM]

Hap_174: 1 [KC330612.1AM]

Hap_175: 1 [KC330611.1AM]

Hap_176: 1 [KC330610.1AM]

Hap_177: 1 [KC330609.1AM]

Hap_178: 1 [KC330608.1AM]

Hap_179: 1 [KC330607.1AM]

Hap_180: 1 [KC330606.1AM]

Hap_181: 1 [KC330598.1AM]

Hap_182: 1 [KC330597.1AM]

Hap_183: 1 [KC330596.1AM]

Hap_184: 1 [KC330595.1AM]

Hap_185: 1 [KC330594.1AM]

Hap_186: 1 [KC330592.1AM]

Hap_187: 1 [KC330591.1AM]

Hap_188: 1 [KC330590.1AM]

Hap_189: 1 [KC330589.1AM]

Hap_190: 1 [KC330587.1AM]

Hap_191: 1 [KC330586.1AM]

Hap_192: 1 [KC330585.1AM]

Hap_193: 1 [KC330584.1AM]

Hap_194: 1 [KC330583.1AM]

Hap_195: 1 [KC330582.1AM]

Hap_196: 1 [KC330581.1AM]

Hap_197: 1 [KC330579.1AM]

Hap_198: 1 [KC330578.1AM]

Hap_199: 1 [KC330577.1AM]

Hap_200: 1 [KC330575.1AM]

Hap_201: 1 [KC330574.1AM]

Hap_202: 1 [KC330573.1AM]

Hap_203: 1 [KC330571.1AM]

Hap_204: 1 [KC330570.1AM]

Hap_205: 1 [KC330569.1AM]

Hap_206: 1 [KC330568.1AM]

Hap_207: 1 [KC330567.1AM]

Hap_208: 1 [KC330566.1AM]

Hap_209: 1 [KC330565.1AM]

Hap_210: 1 [KC330564.1AM]

Hap_211: 2 [KC330560.1AM KC330559.1AM]

Hap_212: 2 [KF668368.1AM KF668403.1AM]

Hap_213: 1 [KF668438.1AM]

Hap_214: 1 [KF668441.1AM]

Hap_215: 1 [AY791551.1AM]

Hap_216: 1 [AY791541.1AM]

Hap_217: 1 [KF668373.1AM]

Hap_218: 2 [KF668390.1AM KF668417.1AM]

Hap_219: 1 [KF668396.1AM]

Hap_220: 1 [KF668402.1AM]

Hap_221: 1 [KF668385.1AM]

Hap_222: 7 [JQ240395.1Guizhou JQ240384.1Guizhou JQ240380.1Guizhou JQ240379.1Guizhou JQ240370.1Guizhou JQ240369.1Guizhou JQ240397.1SE]

Hap_223: 13 [JQ240394.1Guizhou JQ240393.1Guizhou JQ240390.1Guizhou JQ240389.1Guizhou JQ240388.1Guizhou JQ240386.1Guizhou JQ240385.1Guizhou JQ240383.1Guizhou JQ240382.1Guizhou JQ240378.1Guizhou JQ240377.1Guizhou JQ240376.1Guizhou JQ240375.1Guizhou]

Hap_224: 20 [JQ240392.1Guizhou JQ240381.1Guizhou JQ240374.1Guizhou JQ240373.1Guizhou JQ240371.1Guizhou JQ240366.1Anhui JQ240363.1Anhui JQ240362.1Anhui JQ240361.1Anhui JQ240357.1Anhui JQ240356.1Anhui JQ240355.1Anhui JQ240354.1Anhui JQ240352.1Anhui JQ240350.1Anhui JQ240349.1Anhui JQ240347.1Anhui JQ240343.1Anhui JQ240338.1Anhui JQ240333.1Anhui]

Hap_225: 1 [JQ240391.1Guizhou]

Hap_226: 1 [JQ240387.1Guizhou]

Hap_227: 10 [JQ240372.1Guizhou JQ240365.1Anhui JQ240359.1Anhui JQ240358.1Anhui JQ240340.1Anhui JQ240339.1Anhui JQ240337.1Anhui JQ240335.1Anhui JQ240334.1Anhui JQ240398.1SE]

Hap_228: 1 [JQ240368.1Guizhou]

Hap_229: 2 [JQ240367.1Guizhou JQ240351.1Anhui]

Hap_230: 4 [JQ240364.1Anhui JQ240344.1Anhui JQ240336.1Anhui JQ240331.1Anhui]

Hap_231: 1 [JQ240360.1Anhui]

Hap_232: 1 [JQ240353.1Anhui]

Hap_233: 1 [JQ240348.1Anhui]

Hap_234: 1 [JQ240346.1Anhui]

Hap_235: 1 [JQ240345.1Anhui]

Hap_236: 1 [JQ240342.1Anhui]

Hap_237: 2 [JQ240341.1Anhui JQ240332.1Anhui]

Hap_238: 13 [OP250994.1 Hainan OP320684.1 Hainan op320685.1 Hainan OP320687.1 Hainan OP320688.1 Hainan OP320689.1 Hainan OP320690.1 Hainan OP320691.1 Hainan AY598107.1SE AY598105.1SE AY598104.1SE AY598098.1SE AY598063.1SE]

Hap_239: 2 [OP250995.1 Hainan OP320692.1 Hainan]

Hap_240: 1 [OP250996.1 Hainan]

Hap_241: 3 [OP250997.1 Hainan OP320697.1 Hainan]

Hap_242: 1 [OP250998.1 Hainan]

Hap_243: 2 [OP250999.1 Hainan OP320696.1 Hainan]

Hap_244: 6 [OP251000.1 Hainan OP320700.1 Hainan OP320704.1 Hainan OP320706.1 Hainan op250985.1SE op320701.1Ot]

Hap_245: 1 [AY791606.1Hainan]

Hap_246: 5 [OP251001.1 Hainan OP320703.1 Hainan OP320705.1 Hainan OP320707.1 Hainan OP320708.1 Hainan]

Hap_247: 2 [OP251002.1 Hainan OP251004.1 Hainan ]

Hap_248: 2 [OP251003.1 Hainan OP320702.1 Hainan ]

Hap_249: 5 [AB550270.1South AB550271.1South AB550272.1South AB550273.1South AB550275.1South]

Hap_250: 1 [AB550274.1South]

Hap_251: 4 [AB550276.1South AB550277.1South AB550278.1South AB550279.1South]

Hap_252: 1 [AB550280.1South]

Hap_253: 1 [AY791554.1North]

Hap_254: 1 [KC330513.1Korean]

Hap_255: 1 [KC330512.1Korean]

Hap_256: 1 [KC330511.1Korean]

Hap_257: 2 [KC330510.1Korean KC330509.1Korean]

Hap_258: 1 [KC330508.1Korean]

Hap_259: 1 [KC330507.1Korean]

Hap_260: 2 [KC330506.1Korean KC330505.1Korean]

Hap_261: 1 [KC330504.1Korean]

Hap_262: 1 [KC330503.1Korean]

Hap_263: 2 [KC330502.1Korean KC330501.1Korean]

Hap_264: 2 [KC330500.1Korean KC330499.1Korean]

Hap_265: 1 [KC330498.1Korean]

Hap_266: 1 [KC330497.1Korean]

Hap_267: 1 [KF668404.1Korean]

Hap_268: 1 [AY791569.1Oc]

Hap_269: 1 [AY791568.1Oc]

Hap_270: 1 [AY791566.1Oc]

Hap_271: 3 [AY791565.1Oc AY791640.1Oc AY791626.1SW]

Hap_272: 3 [AY791564.1Oc AY791636.1Oc AY791664.1Oc]

Hap_273: 1 [AY791561.1Oc]

Hap_274: 1 [AY791574.1Oc]

Hap_275: 1 [AY791659.1Oc]

Hap_276: 2 [AY791658.1Oc AY791679.1Oc]

Hap_277: 1 [AY791657.1Oc]

Hap_278: 1 [AY791656.1Oc]

Hap_279: 1 [AY791655.1Oc]

Hap_280: 1 [AY791654.1Oc]

Hap_281: 1 [AY791653.1Oc]

Hap_282: 1 [AY791652.1Oc]

Hap_283: 1 [AY791651.1Oc]

Hap_284: 1 [AY791650.1Oc]

Hap_285: 1 [AY791649.1Oc]

Hap_286: 1 [AY791648.1Oc]

Hap_287: 1 [AY791647.1Oc]

Hap_288: 1 [AY791646.1Oc]

Hap_289: 1 [AY791645.1Oc]

Hap_290: 2 [AY791644.1Oc AY791683.1Oc]

Hap_291: 1 [AY791643.1Oc]

Hap_292: 2 [AY791642.1Oc AY791682.1Oc]

Hap_293: 1 [AY791641.1Oc]

Hap_294: 1 [AY791639.1Oc]

Hap_295: 3 [AY791638.1Oc AY791637.1Oc AY791660.1Oc]

Hap_296: 1 [AY791635.1Oc]

Hap_297: 1 [AY791634.1Oc]

Hap_298: 1 [AY791633.1Oc]

Hap_299: 1 [AY791632.1Oc]

Hap_300: 1 [AY791631.1Oc]

Hap_301: 1 [AY791690.1Oc]

Hap_302: 1 [AY791689.1Oc]

Hap_303: 1 [AY791688.1Oc]

Hap_304: 1 [AY791687.1Oc]

Hap_305: 2 [AY791686.1Oc AY791684.1Oc]

Hap_306: 1 [AY791685.1Oc]

Hap_307: 2 [AY791681.1Oc AY791667.1Oc]

Hap_308: 1 [AY791680.1Oc]

Hap_309: 1 [AY791678.1Oc]

Hap_310: 1 [AY791677.1Oc]

Hap_311: 1 [AY791676.1Oc]

Hap_312: 1 [AY791675.1Oc]

Hap_313: 1 [AY791674.1Oc]

Hap_314: 1 [AY791672.1Oc]

Hap_315: 1 [AY791671.1Oc]

Hap_316: 1 [AY791670.1Oc]

Hap_317: 1 [AY791669.1Oc]

Hap_318: 1 [AY791668.1Oc]

Hap_319: 1 [AY791666.1Oc]

Hap_320: 1 [AY791665.1Oc]

Hap_321: 1 [AY791663.1Oc]

Hap_322: 1 [AY791662.1Oc]

Hap_323: 1 [AY791661.1Oc]

Hap_324: 3 [KY923424.1Oc KY923416.1Oc KY923414.1Oc]

Hap_325: 24 [KY923423.1Oc KY923422.1Oc KY923421.1Oc KY923418.1Oc KY923417.1Oc KY923415.1Oc KY923409.1Oc KY923408.1Oc KY923407.1Oc KY923406.1Oc KY923404.1Oc KY923403.1Oc KY923402.1Oc KY923399.1Oc KY923398.1Oc KY923397.1Oc KY923395.1Oc KY923392.1Oc KY923390.1Oc KY923388.1Oc KY923386.1Oc KY923381.1Oc KY923376.1Oc KY923375.1Oc]

Hap_326: 1 [KY923420.1Oc]

Hap_327: 1 [KY923419.1Oc]

Hap_328: 11 [KY923413.1Oc KY923411.1Oc KY923410.1Oc KY923405.1Oc KY923400.1Oc KY923396.1Oc KY923389.1Oc KY923383.1Oc KY923380.1Oc KY923379.1Oc KY923378.1Oc]

Hap_329: 1 [KY923412.1Oc]

Hap_330: 1 [KY923401.1Oc]

Hap_331: 1 [KY923394.1Oc]

Hap_332: 1 [KY923393.1Oc]

Hap_333: 1 [KY923391.1Oc]

Hap_334: 2 [KY923387.1Oc KY923384.1Oc]

Hap_335: 1 [KY923385.1Oc]

Hap_336: 1 [KY923382.1Oc]

Hap_337: 1 [KY923377.1Oc]

Hap_338: 1 [KY923374.1Oc]

Hap_339: 1 [KY923373.1Oc]

Hap_340: 1 [AY598119.1Oc]

Hap_341: 1 [AY598118.1Oc]

Hap_342: 2 [AY598117.1Oc AY598114.1SW]

Hap_343: 1 [AY598116.1Oc]

Hap_344: 1 [KF668387.1Oc]

Hap_345: 2 [KF668391.1Oc op250989.1Oc]

Hap_346: 1 [AY791570.1Oc]

Hap_347: 1 [KF668410.1Oc]

Hap_348: 1 [AY791692.1Oc]

Hap_349: 1 [KC330439.1SW]

Hap_350: 1 [KC330438.1SW]

Hap_351: 1 [KC330437.1SW]

Hap_352: 1 [KC330435.1SW]

Hap_353: 1 [KC330433.1SW]

Hap_354: 1 [KC330432.1SW]

Hap_355: 1 [KC330431.1SW]

Hap_356: 1 [KC330430.1SW]

Hap_357: 2 [KC330429.1SW KC330390.1SW]

Hap_358: 1 [KC330427.1SW]

Hap_359: 1 [KC330426.1SW]

Hap_360: 1 [KC330425.1SW]

Hap_361: 1 [KC330424.1SW]

Hap_362: 1 [KC330423.1SW]

Hap_363: 1 [KC330422.1SW]

Hap_364: 2 [KC330421.1SW KC330386.1SW]

Hap_365: 1 [KC330420.1SW]

Hap_366: 1 [KC330419.1SW]

Hap_367: 1 [KC330418.1SW]

Hap_368: 1 [KC330417.1SW]

Hap_369: 1 [KC330416.1SW]

Hap_370: 1 [KC330415.1SW]

Hap_371: 1 [KC330414.1SW]

Hap_372: 2 [KC330413.1SW KC330387.1SW]

Hap_373: 1 [KC330411.1SW]

Hap_374: 1 [KC330410.1SW]

Hap_375: 1 [KC330409.1SW]

Hap_376: 1 [KC330408.1SW]

Hap_377: 1 [KC330407.1SW]

Hap_378: 1 [KC330406.1SW]

Hap_379: 1 [KC330405.1SW]

Hap_380: 1 [KC330404.1SW]

Hap_381: 1 [KC330402.1SW]

Hap_382: 1 [KC330401.1SW]

Hap_383: 1 [KC330400.1SW]

Hap_384: 1 [KC330399.1SW]

Hap_385: 1 [KC330398.1SW]

Hap_386: 1 [KC330397.1SW]

Hap_387: 1 [KC330396.1SW]

Hap_388: 1 [KC330395.1SW]

Hap_389: 1 [KC330394.1SW]

Hap_390: 1 [KC330393.1SW]

Hap_391: 1 [KC330391.1SW]

Hap_392: 1 [KC330496.1SW]

Hap_393: 1 [KC330495.1SW]

Hap_394: 1 [KC330494.1SW]

Hap_395: 1 [KC330493.1SW]

Hap_396: 1 [KC330492.1SW]

Hap_397: 1 [KC330491.1SW]

Hap_398: 1 [KC330490.1SW]

Hap_399: 1 [KC330489.1SW]

Hap_400: 1 [KC330488.1SW]

Hap_401: 1 [KC330487.1SW]

Hap_402: 1 [KC330486.1SW]

Hap_403: 1 [KC330485.1SW]

Hap_404: 2 [KC330484.1SW KC330483.1SW]

Hap_405: 1 [KC330482.1SW]

Hap_406: 1 [KC330481.1SW]

Hap_407: 1 [KF668407.1SW]

Hap_408: 1 [KF668413.1SW]

Hap_409: 7 [KF668424.1SW AY598115.1SW AY598111.1SW AY598124.1SE AY598123.1SE AY598122.1SE AY598059.1SE]

Hap_410: 2 [KF668427.1SW KF668428.1SW]

Hap_411: 1 [AY791557.1SW]

Hap_412: 1 [AY791575.1SW]

Hap_413: 7 [AY598113.1SW AY598109.1SW AY598071.1SE AY598054.1SE AY598049.1SE AY598040.1SE AY598038.1SE]

Hap_414: 1 [AY598112.1SW]

Hap_415: 1 [AY598110.1SW]

Hap_416: 2 [JN788776.1SW JN788769.1SW]

Hap_417: 3 [JN788775.1SW JN788772.1SW JN788768.1SW]

Hap_418: 3 [JN788774.1SW JN788773.1SW JN788771.1SW]

Hap_419: 1 [JN788770.1SW]

Hap_420: 1 [JN788767.1SW]

Hap_421: 1 [AY791630.1SW]

Hap_422: 1 [AY791629.1SW]

Hap_423: 1 [AY791628.1SW]

Hap_424: 1 [AY791627.1SW]

Hap_425: 1 [AY791625.1SW]

Hap_426: 1 [AY791624.1SW]

Hap_427: 1 [AY791623.1SW]

Hap_428: 1 [AY791622.1SW]

Hap_429: 1 [AY791621.1SW]

Hap_430: 1 [AY791620.1SW]

Hap_431: 1 [AY791619.1SW]

Hap_432: 1 [AY791618.1SW]

Hap_433: 1 [AY791617.1SW]

Hap_434: 2 [AY791616.1SW AY791607.1SW]

Hap_435: 1 [AY791615.1SW]

Hap_436: 1 [AY791614.1SW]

Hap_437: 1 [AY791613.1SW]

Hap_438: 1 [AY791612.1SW]

Hap_439: 1 [AY791611.1SW]

Hap_440: 1 [AY791610.1SW]

Hap_441: 1 [AY791609.1SW]

Hap_442: 1 [AY791608.1SW]

Hap_443: 1 [AY791691.1SW]

Hap_444: 1 [KF668379.1SW]

Hap_445: 1 [OP320699.SW]

Hap_446: 1 [AY791567.1SE]

Hap_447: 1 [AY791563.1SE]

Hap_448: 2 [AY791562.1SE AY791577.1SE]

Hap_449: 1 [AY791560.1SE]

Hap_450: 1 [AY791559.1SE]

Hap_451: 1 [AY791558.1SE]

Hap_452: 1 [AY791556.1SE]

Hap_453: 1 [AY791555.1SE]

Hap_454: 1 [AY791553.1SE]

Hap_455: 1 [AY791552.1SE]

Hap_456: 1 [AY791584.1SE]

Hap_457: 1 [AY791583.1SE]

Hap_458: 1 [AY791582.1SE]

Hap_459: 1 [AY791581.1SE]

Hap_460: 1 [AY791580.1SE]

Hap_461: 1 [AY791579.1SE]

Hap_462: 1 [AY791578.1SE]

Hap_463: 1 [AY791576.1SE]

Hap_464: 1 [AY791572.1SE]

Hap_465: 1 [AY791571.1SE]

Hap_466: 1 [DQ396548.1SE]

Hap_467: 1 [DQ396547.1SE]

Hap_468: 2 [JQ240405.1SE JQ240406.1SE]

Hap_469: 1 [JQ240429.1SE]

Hap_470: 1 [JQ240428.1SE]

Hap_471: 1 [JQ240427.1SE]

Hap_472: 6 [JQ240426.1SE JQ240423.1SE JQ240419.1SE JQ240418.1SE JQ240414.1SE JQ240411.1SE]

Hap_473: 1 [JQ240425.1SE]

Hap_474: 1 [JQ240424.1SE]

Hap_475: 1 [JQ240422.1SE]

Hap_476: 2 [JQ240421.1SE JQ240412.1SE]

Hap_477: 2 [JQ240420.1SE JQ240408.1SE]

Hap_478: 1 [JQ240417.1SE]

Hap_479: 1 [JQ240416.1SE]

Hap_480: 1 [JQ240415.1SE]

Hap_481: 1 [JQ240413.1SE]

Hap_482: 1 [JQ240410.1SE]

Hap_483: 1 [JQ240409.1SE]

Hap_484: 1 [JQ240407.1SE]

Hap_485: 1 [JQ240404.1SE]

Hap_486: 1 [JQ240403.1SE]

Hap_487: 1 [JQ240402.1SE]

Hap_488: 1 [JQ240401.1SE]

Hap_489: 1 [JQ240400.1SE]

Hap_490: 1 [JQ240399.1SE]

Hap_491: 1 [JQ240396.1SE]

Hap_492: 5 [AY598127.1SE AY598053.1SE AY598044.1SE AY598035.1SE op250992.1SE]

Hap_493: 1 [AY598126.1SE]

Hap_494: 1 [AY598125.1SE]

Hap_495: 1 [AY598121.1SE]

Hap_496: 1 [AY598108.1SE]

Hap_497: 1 [AY598106.1SE]

Hap_498: 1 [AY598103.1SE]

Hap_499: 1 [AY598102.1SE]

Hap_500: 1 [AY598101.1SE]

Hap_501: 1 [AY598100.1SE]

Hap_502: 1 [AY598099.1SE]

Hap_503: 2 [AY598073.1SE AY598050.1SE]

Hap_504: 1 [AY598072.1SE]

Hap_505: 1 [AY598070.1SE]

Hap_506: 1 [AY598069.1SE]

Hap_507: 1 [AY598068.1SE]

Hap_508: 1 [AY598067.1SE]

Hap_509: 1 [AY598066.1SE]

Hap_510: 1 [AY598065.1SE]

Hap_511: 1 [AY598064.1SE]

Hap_512: 2 [AY598062.1SE AY598052.1SE]

Hap_513: 1 [AY598061.1SE]

Hap_514: 1 [AY598060.1SE]

Hap_515: 1 [AY598058.1SE]

Hap_516: 1 [AY598057.1SE]

Hap_517: 2 [AY598056.1SE AY598043.1SE]

Hap_518: 1 [AY598055.1SE]

Hap_519: 2 [AY598051.1SE AY598048.1SE]

Hap_520: 3 [AY598047.1SE AY598046.1SE AY598042.1SE]

Hap_521: 1 [AY598045.1SE]

Hap_522: 1 [AY598041.1SE]

Hap_523: 1 [AY598039.1SE]

Hap_524: 1 [AY598037.1SE]

Hap_525: 1 [AY598036.1SE]

Hap_526: 1 [KC330557.1SE]

Hap_527: 3 [KC330556.1SE KC330555.1SE KC330554.1SE]

Hap_528: 1 [KC330553.1SE]

Hap_529: 2 [KC330552.1SE KC330550.1SE]

Hap_530: 1 [KC330551.1SE]

Hap_531: 1 [KC330549.1SE]

Hap_532: 1 [KC330548.1SE]

Hap_533: 1 [KC330547.1SE]

Hap_534: 1 [KC330546.1SE]

Hap_535: 1 [KC330545.1SE]

Hap_536: 5 [KC330544.1SE KC330541.1SE KC330540.1SE KC330539.1SE KC330538.1SE]

Hap_537: 1 [KC330543.1SE]

Hap_538: 1 [KC330542.1SE]

Hap_539: 1 [KC330537.1SE]

Hap_540: 1 [KC330536.1SE]

Hap_541: 1 [KC330535.1SE]

Hap_542: 2 [KC330534.1SE KC330533.1SE]

Hap_543: 2 [KC330532.1SE KC330531.1SE]

Hap_544: 1 [KC330530.1SE]

Hap_545: 1 [KC330529.1SE]

Hap_546: 1 [KC330528.1SE]

Hap_547: 1 [KC330527.1SE]

Hap_548: 1 [KC330526.1SE]

Hap_549: 1 [KC330525.1SE]

Hap_550: 1 [KC330524.1SE]

Hap_551: 1 [KC330523.1SE]

Hap_552: 2 [KC330522.1SE KC330519.1SE]

Hap_553: 1 [KC330521.1SE]

Hap_554: 1 [KC330520.1SE]

Hap_555: 1 [KC330518.1SE]

Hap_556: 1 [KC330517.1SE]

Hap_557: 1 [KC330516.1SE]

Hap_558: 1 [KC330515.1SE]

Hap_559: 1 [KC330514.1SE]

Hap_560: 1 [DQ396549.1SE]

Hap_561: 1 [AY791573.1SE]

Hap_562: 1 [KF668400.1SE]

Hap_563: 2 [op250986.1SE op320686.1SE]

Hap_564: 1 [op250987.1SE]

Hap_565: 1 [op250993.1SE]

Hap_566: 1 [AY791599.1Ot]

Hap_567: 2 [AY791598.1Ot AY791596.1Ot]

Hap_568: 1 [AY791597.1Ot]

Hap_569: 1 [AY791595.1Ot]

Hap_570: 1 [AY791594.1Ot]

Hap_571: 1 [AY791593.1Ot]

Hap_572: 1 [AY791592.1Ot]

Hap_573: 1 [AY791591.1Ot]

Hap_574: 1 [AY791590.1Ot]

Hap_575: 1 [AY791589.1Ot]

Hap_576: 1 [AY791588.1Ot]

Hap_577: 1 [AY791605.1Ot]

Hap_578: 1 [AY791604.1Ot]

Hap_579: 1 [AY791603.1Ot]

Hap_580: 1 [AY791602.1Ot]

Hap_581: 1 [AY791601.1Ot]

Hap_582: 1 [AY791600.1Ot]

Hap_583: 3 [AY598139.1Ot AY598133.1Ot AY598132.1Ot]

Hap_584: 2 [AY598138.1Ot AY598135.1Ot]

Hap_585: 3 [AY598137.1Ot AY598130.1Ot AY598129.1Ot]

Hap_586: 1 [AY598136.1Ot]

Hap_587: 1 [AY598134.1Ot]

Hap_588: 1 [AY791587.1Ot]

Hap_589: 1 [AY791586.1Ot]

Hap_590: 1 [AY791585.1Ot]

Hap_591: 1 [AY598131.1Ot]

Hap_592: 1 [AY598128.1Ot]
